# Rathke cleft cyst masquerading as pituitary abscess: A case report

**DOI:** 10.1097/MD.0000000000006303

**Published:** 2017-03-10

**Authors:** Chengxian Yang, Xinjie Bao, Xiaohai Liu, Kan Deng, Ming Feng, Yong Yao, Renzhi Wang

**Affiliations:** Department of Neurosurgery, Peking Union Medical College Hospital, Peking Union Medical College & Chinese Academy of Medical Sciences, Beijing, China.

**Keywords:** case report, pituitary abscess, Rathke cleft cyst, surgery

## Abstract

**Background:**

Rathke cleft cyst (RCC) is a rare cystic sellar entity, which is usually small in size and asymptomatic in most patients. RCC presenting panhypopituitarism and a cystic lesion with rim enhancement on magnetic resonance imaging is extremely rare. Therefore, it is easy to be misdiagnosed as pituitary abscess because of the similar clinical manifestations and neuroimaging changes.

**Case summary:**

We report a rare case of RCC masquerading as pituitary abscess clinically and radiologically with no evidence of central nervous system infection. The patient was initially suspected to be diagnosed with pituitary abscess, which was denied by the histopathological findings of RCC with no intraoperative drainage of abscess.

We present an uncommon case of RCC masquerading as pituitary abscess in a 62-year-old Chinese male patient. The patient was admitted to Peking Union Medical College Hospital complaining of severe frontal pulsatile headache, visual acuity deficit, polyuria, polydipsia, and slight disturbance of consciousness. The biochemical and endocrinological examinations revealed severe hyponatremia and panhypopituitarism. Magnetic resonance imaging showed a sellar lesion with the apparent cystic change and rim enhancement. Accordingly, pituitary abscess was misdiagnosed at the beginning.

The patient received hormone replacement therapy and underwent a trans-sphenoidal surgery. The surgical findings were uneventful. The histopathological examinations showed no infiltration of inflammatory cells or pus, and proved the lesion to be RCC.

**Conclusion:**

Through this rare case, we aim to emphasize that the differential diagnosis of sellar lesions requires constant vigilance and that RCC may lead to clinical and radiological changes similar with pituitary abscess.

## 1 Introduction

Rathke cleft cyst (RCC) is an uncommon cystic sellar and suprasellar entity that originates from the residuals of Rathke pouch, which is lined with the cuboidal or columnar epithelium. RCC is frequently detected on post mortem examinations with a rate of 13% to 22%, because it is commonly small in size and asymptomatic without mass effect or endocrine impairment in most patients.^[1,2]^ Pituitary abscess, which accounts for <1% of the pituitary lesions, is also an uncommon but life-threatening disease characterized by endocrine impairment, mass effect, and typical magnetic resonance imaging (MRI) features.^[3–5]^ Pituitary abscess can be secondary to a healthy pituitary gland or an existed sellar lesion such as pituitary adenoma, craniopharyngioma, and RCC. Herein, we present an extremely rare patient diagnosed with RCC mimicking pituitary abscess.

## 2 Case report

**Figure 1:**
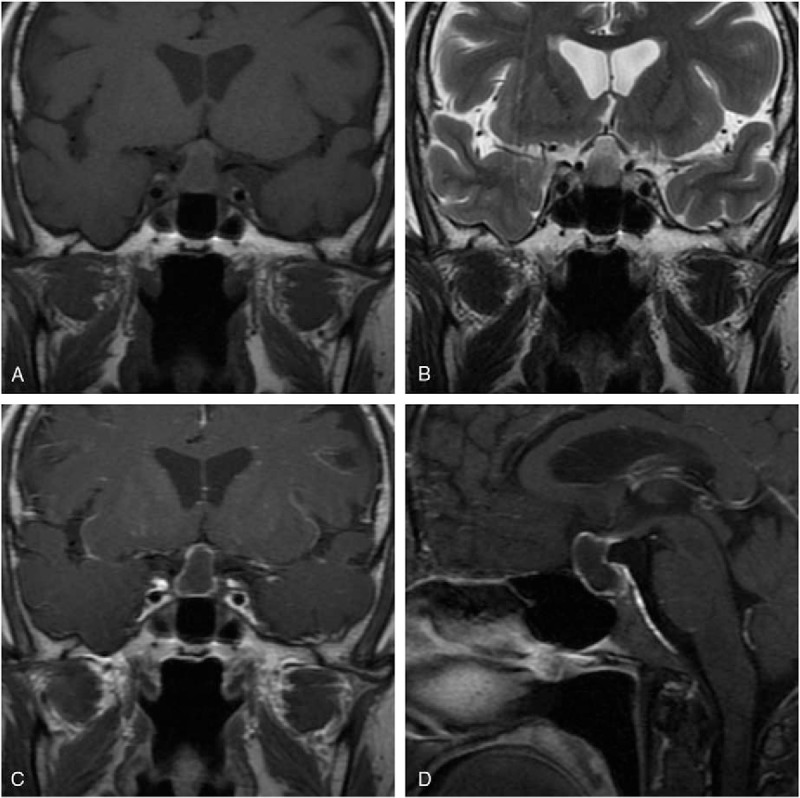
Preoperative MRI of showed the sellar and suprasellar entity. (A) Coronal T1WI; (B) coronal T2WI; (C) coronal-enhanced T1WI; (D) sagittal-enhanced T1WI. MRI = magnetic resonance imaging, T1WI = T1-weighted image, T2WI = T2-weighted image.

**Figure 2:**
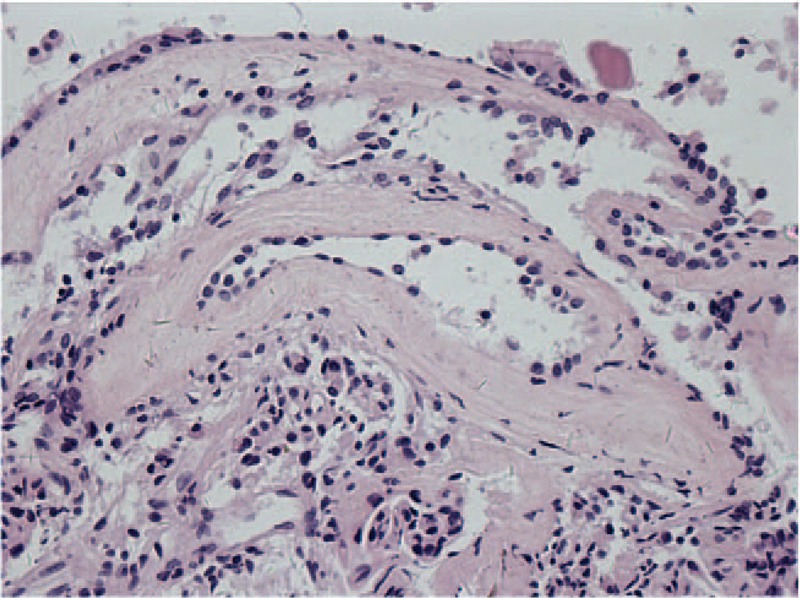
Histopathological examination of the lesion (hematoxylin and eosin staining ×400) showed the simple cuboidal epithelial cell lining of the cyst proving the lesion to be RCC. RCC = Rathke cleft cyst.

**Figure 3:**
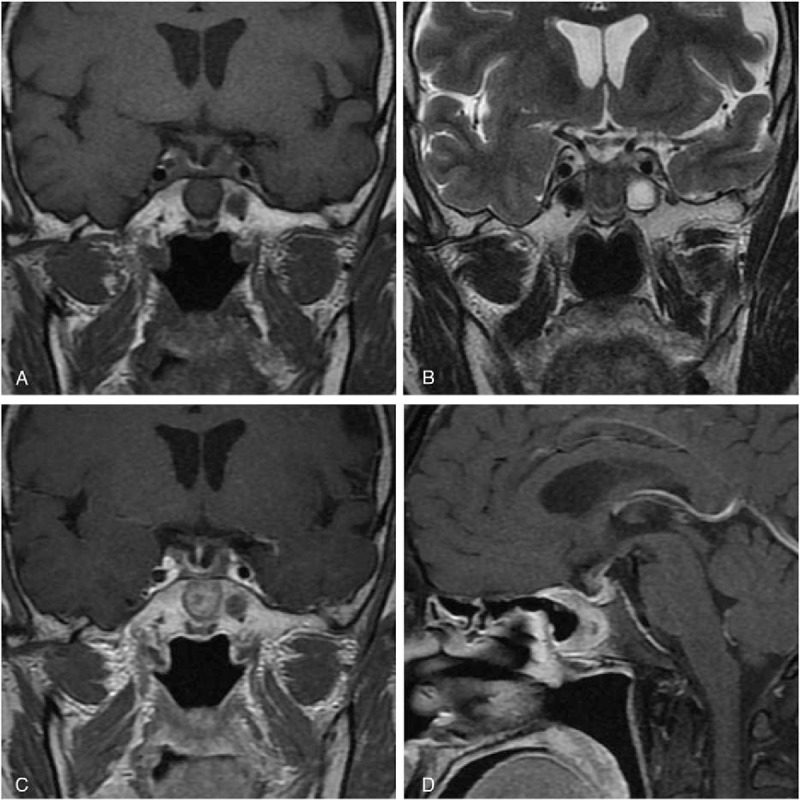
Postoperative MRI of showed the lesion after 3- month follow-up. (A) Coronal T1WI; (B) coronal T2WI; (C) coronal-enhanced T1WI; (D) sagittal-enhanced T1WI. MRI = magnetic resonance imaging, T1WI = T1-weighted image, T2WI = T2-weighted image.

A 62-year-old Chinese male patient was admitted to Peking Union Medical College Hospital (PUMCH) complaining of a 6-month history of frontal chronic headache, which used to be alleviated by nonsteroidal anti-inflammatory drugs or rest, from September 2014. The pain was intermittent, pulsatile, and moderate, and occurred about 3 times every month in the previous 5 months. The patient did not seek for medical services until the pain increased in intensity and frequency from the end of February 2015. The results of hormone work-up were uneventful. Brain MRI scan with contrast was performed in the local hospital showing a round-like cystic lesion measuring approximate 20 mm in diameter in sellar region with homogeneous signal characteristics and ring enhancement. The diagnosis of RCC was initially made. However, the patient started to have precipitously worsening conditions including severe frontal pulsatile headache, visual acuity (VA) deficit of the right side, polyuria (>5000 mL/24 h), polydipsia, and even slight disturbance of consciousness. He was transferred to our hospital by ambulance in the middle of March 2015. The patient did not present with nausea, vomiting, high temperature, and other symptoms related to central nervous system infection. The previous history of paranasal sinus infection or meningitis was negative in our patient. He did not report the regular or previous intake of immunosuppressive drugs. His medical history was otherwise unremarkable. The general physical examination was uneventful. The neurologic examination demonstrated that the patient was awake, alert, and oriented to self, time, and location. The limb muscular strength and tension of our patient were normal. The signs of meningeal irritation were completely negative. Various physiological reflexes existed. The pathological signs were absent. The results of ophthalmologic test indicated mild decline of VA of his right eye and no remarkable loss of the visual field. Preoperatively, VA of the right eye was 0.6. Results of hematologic tests were uneventful. Electrolytes examinations revealed severe hyponatremia (Na: 118 mmol/L; reference range: 135–145 mmol/L). Before surgery, correction of hyponatremia (Na: 147 mmol/L) was done with hypertonic saline. Endocrine evaluations showed hyperprolactinemia and severe panhypopituitarism requiring instant hormone replacement therapy (Table 1). MRI demonstrated a sellar and suprasellar lesion with iso-hypointensity, with peripheral enhancement after administration of gadolinium-diethylene triamine pentaacetic acid (Gd-DTPA) on T1-weighted images (T1WI) and iso-hyperintensity on T2-weighted images (T2WI) measuring 18 mm × 14 mm × 20 mm. These imaging characteristics were of diagnostic value pointing to pituitary abscess (Fig. 1). The patient was diagnosed with pituitary abscess based on the clinical, biochemical, and imaging features. A trans-sphenoidal procedure was performed. In the procedure, purulent materials did not occur as we had expected before the operation. Yellowish-brown homogeneous materials were detected and completely drained out. The cavity was irrigated with saline, and no residual tissues were found. Neither leakage of cerebrospinal fluid (CSF) nor intracranial infection happened during the procedure. RCC was pathologically confirmed (Fig. 2). In the postoperative period, cefuroxime (1500 mg qd) was administered intravenously for 3 days. In terms of hormone replacement, the patient was treated with hydrocortisone (100 mg q12 hours) intravenously for 3 days and then prednisone (20 mg q12 hours) orally and levothyroxine (100μg qd). Desmopressin acetate tablets (0.1 mg q12 hours) were taken orally to control diabetes insipidus. Blood sodium remained normal and ranged 140 to 153 mmol/L. The ophthalmologic test was not conducted after surgery because the patient complained of no visual changes. The patient's postoperative course was otherwise uneventful. During a 3-month follow-up of our patient, the intensity of headache alleviated significantly and almost disappeared, and in the meantime the impairment of VA markedly abated as well. However, desmopressin acetate tablets were still needed to control the symptoms of polyuria and polydipsia. Hormone replacement therapy continued. The examination of hormone levels indicated the restoration of pituitary endocrine function with the routine oral intake of levothyroxine (100μg qd) and prednisone (10 mg qd8; 5 mg qd16) tapering to a maintenance dosage (2.5 mg qd8) (Table 1). The daily dosage of desmopressin acetate dropped from 0.1 mg q12 hours to 0.05 mg q12 hours, and polyuria was under control (<3000 mL/24 h). MRI with contrast was also conducted showing no residual or recurrent lesions (Fig. 3). During a 12-month follow-up, the quality of life of our patient was further improved with previous routine intake of medicine.

**Table 1:**
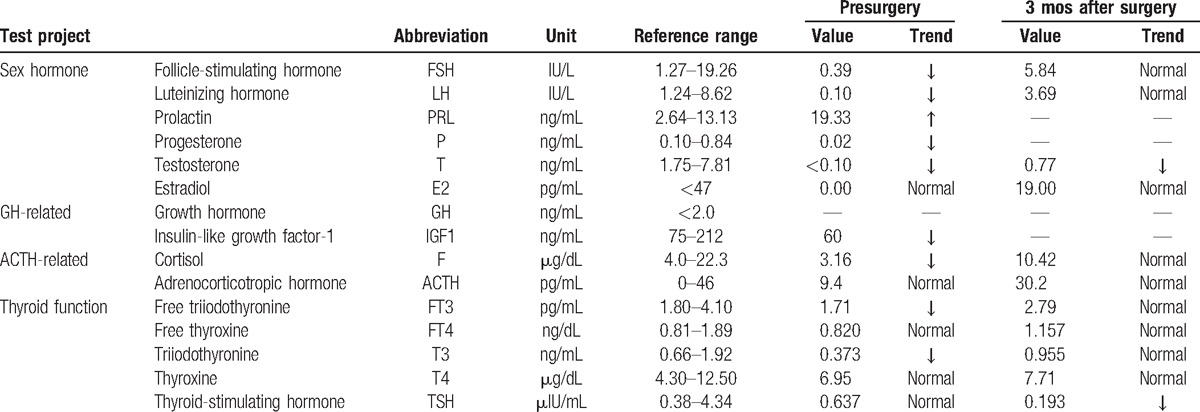
Results of endocrine studies for the pituitary gland before and after surgery.

## 3 Discussion

In the case report, we present a unique and rare case of RCC mimicking pituitary abscess in terms of clinical manifestations and neuroimaging.

Rathke cleft cyst was firstly described in a published case report by Luschka in 1860 and then presented as a symptomatic lesion by Goldzieher in 1913.^[6]^ RCC usually remains asymptomatic throughout the life without mass effect and endocrine impairment. Central diabetes insipidus indicating posterior pituitary gland damage in RCC is quite uncommon and is usually reported in isolated case reports or series with the frequency ranging from 0% to 19%.^[7]^ According to limited published cases, diabetes insipidus in patients with RCC results from the following reasons including atypical location of RCC at the pituitary stalk,^[8]^ stalk impairment,^[9]^ unusual course of rapid growth,^[10]^ primary inflammation,^[11]^ and coexistence with some destructive pituitary lesions.^[12–14]^ Pituitary abscess formation within RCC does not usually result in significant diabetes insipidus.^[15,16]^ Panhypopituitarism is rare in RCC though patients with symptomatic RCC usually have pituitary dysfunction of varying degrees.^[17–19]^ In our case, the endocrine work-up results revealed panhypopituitarism resulting in adrenal insufficiency, hypothyroidism, and gonadal failure. Prompt replacement therapy with levothyroxine and prednisone significantly improved the clinical symptoms in our case. Surgical management is highly recommended to protect the pituitary function and prevent the irreversible panhypopituitarism.^[18]^ Panhypopituitarism is rare in RCC,^[20,21]^ but quite common in pituitary abscess as a result of severe pituitary gland damage according to our own experiences and other case series.^[3,5,22,23]^

Magnetic resonance imaging is the first choice of neuroimaging in the diagnosis of RCC. MRI signal characteristics of RCC vary greatly according the biochemical nature of intracystic material.^[21]^ Though being reported in RCC imaging, the rim enhancement on T1WI with Gd-DTPA is negative in most patients with RCC.^[7,21]^ In contrast, rim enhancement is usually considered relatively specific in pituitary abscess.^[3,22,24,25]^ Compared with conventional T1WI and T2WI, diffusion-weighted image (DWI) shows greater value in distinguishing RCC from other cystic sellar entities.^[26]^ The significant hypointensity of RCC was detected on DWI in contrast to iso to hyperintensity of craniopharyngiomas and hemorrhagic pituitary adenomas.

Pituitary abscess was firstly described by Simmonds in 1914, and then presented in case reports or series.^[27]^ Pituitary abscess is classified into primary and secondary types accounting for 2/3 and 1/3 of its causes, respectively. The current or prior infection serves as an important clue for the diagnosis of pituitary abscess, though infection history is not frequently seen. Among all the published studies, Liu et al^[22]^ from PUMCH represented the largest case series of 33 pituitary abscess between 1991 to 2007, and summarized the experiences in terms of clinical manifestations, pituitary imaging, and biochemical results of pituitary abscess. In most published articles, typical pituitary abscess was described to present with diabetes insipidus, panhypopituitarism, and sellar cystic mass with an enhanced rim on MRI, all of which were quite similar with the symptoms of our patient.

According to relevant case reports, typical content of infected RCC was described as white-yellow or yellow purulent materials different from the drained content in our case.^[15,28]^ The limitation of our case was that routine cytology test and bacterial culture were not conducted, though the pathological test showing no inflammation cell infiltration partially proved the lesion not to be infectious. The clinical progression of our patient mainly included symptomatic deteriorations, severe hyponatremia, and consciousness disturbance. According to our experiences, pituitary dysfunction and potential infection might be the main causes for the progression. Sufficient preoperative preparation was administrated to correct electrolyte disorders and make our patient stable. We think that negative results of inflammation cells attributed to preoperative treatment. Thus, it is hypothesized that the atypical performance of our patient indicated that the lesion was in an early transition phase toward pituitary abscess secondary to infected RCC. The decline of immunity function of unknown origin might accelerate the transition and result in severe deterioration of pituitary function.

Endoscopic endonasal trans-sphenoidal surgery is preferably adopted as the first-line treatment option for such sellar lesion with suspected infection.^[22,23]^ Craniotomy is not routinely recommended as it is associated with a higher risk of infectious content exposure to the adjacent structures.^[3]^ Intraoperative findings and histopathological results remain the gold standard for the diagnosis.

## 4 Conclusions

Pituitary abscess is associated with high mortality. Early detection, prompt hormone replacement, complete abscess drainage, and appropriate antibiotic therapy are the key components of curing pituitary abscess. A cystic mass with rim enhancement on MRI is of diagnostic value, but not specific enough in diagnosing pituitary abscess. Although uncommon, RCC is supposed to be taken into consideration in the differential diagnosis of pituitary abscess. The precise mechanism of RCC without infection showing such clinical and radiographic similarity with pituitary abscess remains unclear and requires further exploration.

## 5 Consent

Ethical approval to report this case was not required. Written informed consent was obtained from our patient for the case report publication. A copy of the written consent is available for review by the Editor of the journal.
